# A CAST-Based Analysis of the Metro Accident That Was Triggered by the Zhengzhou Heavy Rainstorm Disaster

**DOI:** 10.3390/ijerph191710696

**Published:** 2022-08-27

**Authors:** Jiale Zhao, Fuqiang Yang, Yong Guo, Xin Ren

**Affiliations:** 1 College of Environment and Safety Engineering, Fuzhou University, Fuzhou 350116, China; 2 Safety and Security Science Group, Faculty of Technology, Policy and Management, Delft University of Technology, 2628 BX Delft, The Netherlands

**Keywords:** China, emergency management, extreme weather, urban waterlogging, CAST

## Abstract

Emergency management research is used to deal with the increasing number of extreme weather threats in urban areas. This paper uses causal analysis based on systems theory (CAST) to review the subway water ingress accident and the government’s emergency management actions in Zhengzhou, Henan Province, during the heavy rainstorm disaster on 20 July 2021. The aims of this article are to establish safety control structures at both the enterprise level and the government level, and to systematically analyze the problems in emergency management in Zhengzhou City. Our analysis found that the construction of disaster prevention facilities restricted emergency management. Therefore, we suggest that enterprises and governments not only pay attention to emergency management, but also to the construction of disaster prevention facilities. This article also points out that the system of chief executive responsibility that is implemented in China is becoming a double-edged sword in emergency management. Our study makes recommendations for enhancing the capacities of emergency management, points out the shortcomings of the existing emergency management structure, and provides knowledge gained for future emergency management research.

## 1. Introduction

Extreme weather is defined by the World Meteorological Organization as “the occurrence of a value of a weather or climate variable above (or below) a threshold value near the upper (or lower) ends of the range of observed values of the variable”. As human influence on climate increases [1,2,3], extreme weather occurs more frequently [4]. In China, extreme weather, such as extreme heat and heavy rainstorms, is becoming more frequent due to global warming [5]. According to the China Meteorological Administration, the national average temperature in 2021 was 10.5 °C, which is 1.0 °C higher than the national average temperature from1981 to 2010. The national average precipitation in 2021 was 672.1 mm, which was 6.7% more than normal. 

Extreme heat is likely to induce cardiovascular and respiratory diseases, thereby increasing mortality, especially for the elderly [6,7]. Heavy rainstorms increase the risks of flooding and urban waterlogging [8,9]. On 21 July 2012, a heavy rainstorm occurred in Beijing, resulting in 79 deaths and affecting 16.02 million people [10]. In July 2017, a heavy rainstorm in Nanjing caused urban waterlogging [11]. On 11 April 2019, the heavy rainstorm in Shenzhen lasted only 1 h and 42 min [12], but the ensuing urban flood left more than ten people dead in the city. Waterlogging caused by heavy rainstorms has seriously affected the development of large and medium-sized cities in China [13,14,15]. Approximately 62% of the cities in China have experienced urban waterlogging events in recent years, especially in the eastern urban agglomeration [16]. Such events mean that city managers need to conduct more effective disaster emergency management [17].

Scholars from various countries have explored disaster emergency management. For example, the operations research or management science (OR/MS) method was applied to study the problems of resource allocation in the context of a disaster [18]. Since the 1970s, researchers have gradually realized that in addition to studying the physical damage caused by disasters, the social and economic damages caused by disasters are worthy of consideration [19]. After examining extensive literature on organizational management during disasters, Bundy et al. [20] proposed a comprehensive crisis management framework. Sperling et al. [21] suggested using operations research to coordinate the services of spontaneous volunteers during disasters. Research into emergency management initially came from enterprises [22] and the non-profit sector [23]; gradually, government departments became involved [24]. Early warning techniques for disasters, such as flood forecasting, have developed from the traditional physically based models [25] and numerical simulation methods [26] to forecasting on the basis of machine learning [27]. In terms of disaster emergency medical care, after the 2004 Indian Ocean tsunami caused serious casualties, emergency medical responses have been the subject of research throughout the world [18]. The important role of hospitals as medical service centers during and after disasters has been repeatedly addressed [28,29].

Current research on emergency management focuses mainly on the management of accidents [30,31], including the application of statistical methods to study accident characteristics [32], evacuation route planning in emergencies [33,34], accident analysis methods [35,36], emergency response methods [37], hazardous chemical leakage assessment [38,39], risk management [40], and the prediction of accidents with artificial intelligence [41,42]. There are relatively few recent case studies on the emergency management of natural disasters. However, with climate change, extreme weather causes increasing losses for human beings. The conduct of effective emergency management during and after natural disasters is a problem that requires more research attention. 

In recent years, research on disaster emergency management concentrated mainly on using various technical means to prevent disasters, including developing emergency early warning systems [43], applying artificial intelligence for disaster management [44], using big data to analyze disasters [45], and using deep learning methods to predict landslides [46]. There are few scholars using accident investigation methods to review the disasters that have occurred and analyze deficiencies in management. Over the past few decades, China’s emergency management system at the national level has been continuously improved, but the heavy rainstorm disaster in Zhengzhou, Henan Province, on 20 July 2021 [47] revealed that the system has some deficiencies. 

In this study, we hope to identify the deficiencies of the existing emergency management system by analyzing the heavy rainstorm disaster in Zhengzhou and putting forward suggestions to eliminate these deficiencies. This study applies causal analysis based on systems theory (CAST) for the following four reasons. 

(1) An emergency system is a complex system involving many components, and the CAST method is suitable for such a system as it provides a framework for reviewing the system [48]. It can be used to sort out the relationships between system components, changes in the components, and the impact of the changes. Reasonable recommendations may be made after the analysis is completed [49]. 

(2) The CAST approach divides the analysis into different levels, extending from the physical level where the accident occurred to the management level, the government level, and even the level of national legislation. The role of government is a part of the natural disaster emergency management process that cannot be ignored; its importance goes beyond the physical level. Because the government usually coordinates various resources to respond to natural disasters from a macro perspective, the results of a CAST analysis will provide optimized recommendations for the entire structure. 

(3) The CAST approach emphasizes the dynamic characteristics of a system and analyzes the causes of accidents that may occur through the control behavior and action feedback between system components. In contrast, traditional event chain models treat systems as static and unchanging. Therefore, the CAST method is more suitable for analyzing system defects. 

(4) The CAST method places value on communication and consultation within a system. On the one hand, the leader of an emergency management operation must communicate and coordinate with other components of the system to accomplish the goals of the emergency effort. On the other hand, there may be multiple leaders for a single emergency effort, and the CAST approach helps in identifying problems among these leaders. 

For all of these reasons, we used the CAST method to study the emergency management actions during and after the heavy rainstorm disaster in Zhengzhou, Henan Province, from the perspective of system science. Our analysis was conducted at both the company and government levels, with the aim of optimizing the existing emergency management structure for dealing with natural disasters, based on the CAST approach.

## 2. Methodology

Accident models are an essential part of safety science. The traditional accident model is based on the belief that the accident process can be summarized as a chain of events. The event leading to the beginning of the chain of events is regarded as the root cause of the accident [50]. However, such methods tend to blame the operator for the cause of the accident, obscuring the system-level cause [51]. Therefore, accident theories based on systems theories, such as the systems theoretic accident model and process (STAMP) [52,53], AcciMap [54], and the functional resonance analysis method (FRAM) [55], have been developed. These theories hold that with the increasingly complex structure of modern systems, it is difficult to find the so-called “root cause” according to the traditional accident model, and the cause of an accident may be the interactions between several system components [56]. Therefore, modern accident models must consider the relationships between the parts of a system. 

**Figure 1:**
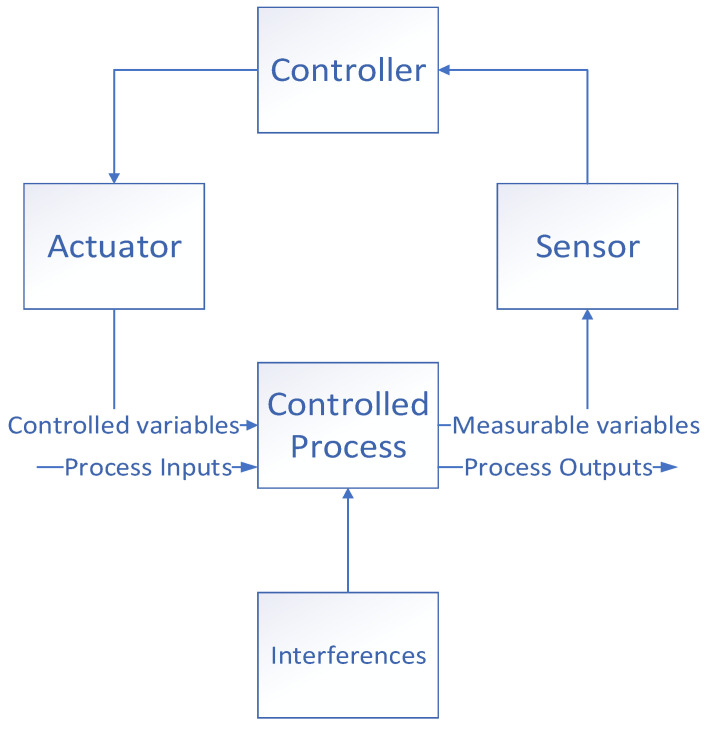
The control loop of the system deals with interferences.

In previous studies, the STAMP model has been proven to be a practical accident analysis theory [57,58,59]. Its theoretical basis comes from system theory and control theory [60]. The basic structure of STAMP includes security constraints, a hierarchical control structure, and a process model [61]. The core idea of STAMP theory is to transform a safety problem into a control problem. The safe operation of a system depends on adding means of control to a system during the design and operation stages to ensure that safety constraints are effectively implemented. These means of control can be physical security components or organizational structure, management, culture, etc. [62]. A hierarchical control structure means that the higher levels in a system exert control over the lower levels to ensure that the security constraints of the lower levels are enforced. The structure is bidirectional. The high-level components issue decisions to the low-level components. The low-level components provide the high-level components with the information needed for decision-making and with feedback after a decision is executed. Process models are derived from cybernetics to show the rules that controllers follow in a specific control process, i.e., what action to take under what circumstances and what following steps to take based on the feedback information. STAMP theory considers conflicts between process models and actual processes as an important cause of accidents [61]. Figure 1 shows a classic control loop: the controller obtains the current parameters of the system through sensors and adjusts the behavior of the actuators according to the parameter changes to ensure that the values of the parameters remain within acceptable limits, even when the system is disturbed. The causal analysis based on systems theory (CAST) method was developed based on the STAMP theory, with the goal of determining the cause of the accident and drawing lessons from it.

The CAST method is a set of workflows that use STAMP theory to analyze accidents. The CAST method investigates accidents by analyzing factors such as context, communication and coordination, control, and mental models, based on the STAMP structure. The “context” is used to analyze the environment in which the system components are located at that time; “communication and coordination” are used to analyze the relationship between the system components and to show it in the form of a diagram; “control” is used to analyze the failed control actions during the accident; the “mental model” is used to analyze the causes of human error in the system [61]. Leveson classified the causes of accidents into four categories: improper controller operation, improper actuator operation, control process failure, and inaccurate, missing, or delayed information feedback (Leveson, 2011). The main contribution of CAST is to provide a process and framework for incident investigation using STAMP theory. The CAST approach can be divided into six steps: establishing a chain of relevant events, determining system safety constraints, establishing a system control structure, carrying out a physical layer analysis, carrying out a high-level control structure analysis, and formulating recommendations. The purpose of CAST is not to determine who should be responsible for an accident, but rather to find the cause of the accident and to identify system defects (Düzgün and Leveson, 2018). This method has been widely applied in accident analysis, including railway accidents [58], shipwrecks [51,63], mining accidents [49], and pipeline gas explosion accidents [64], with the potential to be applied in additional fields and industries.

## 3. CAST Analysis of the Zhengzhou Henan Heavy Rainstorm Disaster

### 3.1. Brief Description of the Disaster

From 17 July 2021, to 23 July 2021, Henan Province in China was hit by a historically rare heavy rainstorm, affecting more than 14 million people in 150 counties (cities and districts). There were 380 fatalities in Zhengzhou, accounting for 95.5% of the total fatalities of Henan Province. Besides, the direct economic loss of Zhengzhou in this disaster reached 40.9 billion RMB, which accounts for 34.1% of that of the whole Henan province. After the disaster, to summarize the lessons learned from this sudden natural disaster, the State Council of the People’s Republic of China set up an investigation team of the heavy rainstorm disaster, with the professional support of experts from various fields. The investigation concluded that “the heavy rainstorm disaster in Zhengzhou, Henan Province, was a particularly significant natural disaster caused by extreme rainstorms that led to severe urban flooding, river flooding, landslides, and other multiple disasters, resulting in significant casualties and property damage.” In addition, the local government’s dereliction of duty and malfeasance in responding to the disaster led to damages that could have been avoided.

During the rainstorm disaster, representative incidents included the fatal incident on Metro Line 5, the fatal incident at the Jingguang Expressway North Tunnel, and the flash flood at Wang Zongdian Village in Cui Miao Town, Xingyang City. In this study, the fatal incident on Metro Line 5 was selected for analysis for the following reasons: (1) it involved the Zhengzhou Metro Group, a corporate level, and the Zhengzhou municipal government, a level of government that has a complete system structure that enables a more representative analysis than is possible for other incidents; and (2) it resulted in a larger number of trapped people, with a greater impact on society.

### 3.2. CAST Analysis

#### 3.2.1. Related Event Chain

The Henan Provincial Meteorological Bureau provided an early warning before the heavy rainstorm. The State Council working group arrived in Zhengzhou and other places two days before the rainstorm to guide the flood control work. On 13 July 2021 and 16 July 2021, the Henan provincial party committee and the provincial government initiated a special response for the flood control work. However, the Zhengzhou municipal party committee and the municipal government did not arrange sufficient deployment for the upcoming rainstorm, but only proposed some conventional measures. They also failed to ensure the effective implementation of flood control measures. It was not until the morning of 20 July 2021, that the municipal government and the other levels of government began to check on flood control. At that time, the meteorological department issued a second red warning for heavy rain. However, the officials did not carry out measures such as the mandatory suspension of classes and businesses, and people continued to go to work and school as normal. This resulted in many people being killed on their way to and from work or school. After the Zhengzhou Metro Group Co., Ltd. (Zhengzhou, China), issued a warning from the meteorological department, it did not conduct a comprehensive investigation of the hidden dangers on the lines it operated, nor did it arrange for the person in charge to take command of the operating control center (OCC). At 4:00 p.m. on 20 July 2021, Metro Line 5 leaked in many places; then, at about 5:00 p.m., a large amount of waterlogged water poured into the metro tunnel. Although the train stopped temporarily at the Beach Temple station, it continued to travel at 5:46 p.m. under conditions that were not fully understood. When the water flooded the tracks, the driver stopped following the regulations, and the train dispatcher instructed the train to retreat. After retreating for about 30 m, the train lost power. At that time, there was water in the train. Then, the subway operation branch did not report the dangerous situation to the head office in a timely manner, and did not commence the emergency response. The chain of events is shown in Table 1.

#### 3.2.2. System Goals and Security Constraints

Security constraints are the means that exist in a system to prevent hazards from occurring [61]. This study describes the security constraints of the emergency management system at the operational, corporate, and governmental levels. 

The security constraints at the operational level included the drivers driving trains according to regulations and reporting any dangerous situations to the OCC on time; the OCC exercising responsibility for dispatching trains and issuing instructions to train drivers to move forward or backward or to pause to ensure safe train operation; and the OCC serving as the metro emergency command center, commanding emergency forces within the metro system to respond to emergencies and report relevant situations to the operating company. 

The security constraints at the company level included company compliance with relevant laws and regulations; the company formulating appropriate emergency plans according to various possible situations and ensuring that employees were familiar with the contents of the plans; the company conducting safety inspections of the subway lines under its management to ensure safe operation; measures to ensure the safe evacuation of train passengers when an emergency occurred; and the company promptly reporting any dangerous situation to the government transportation department. 

The security constraints at the governmental level included the local government formulating various emergency plans for the region; the local government issuing early warnings to the public about predicted dangers; the local government announcing to the citizens the interruption of daily production and life after the danger reached a certain level; the local government directing professional emergency forces to respond to various emergencies; and the local government supervising various security efforts within its jurisdiction. 

After identifying the safety constraints, a safety control structure should be established. In this study, a safety control structure for Zhengzhou Metro will be established for the operational level and the company level, and the Zhengzhou government’s emergency control structure will be established at the government level. All security constraints are shown in Table 2.

#### 3.2.3. The Security Control Structure of Zhengzhou Metro

**Figure 2:**
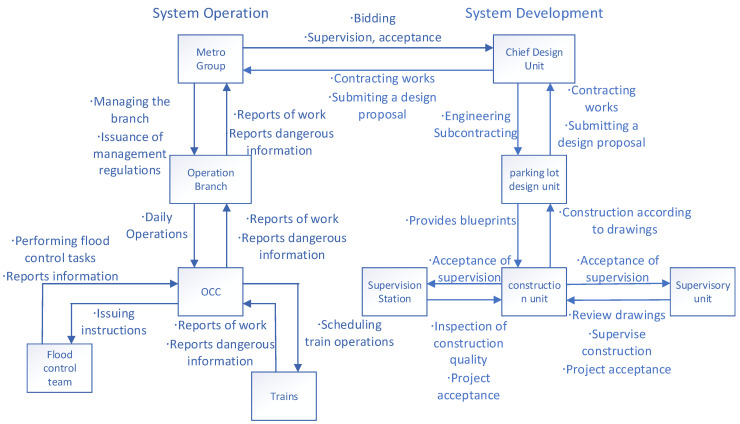
The security control structure for Zhengzhou Metro.

In general, the control structure obtained from the CAST analysis consists of two parts: system development and system operation. The specific control structure is shown in Figure 2.

(1) System operation

The leading unit managing Zhengzhou Metro is the Zhengzhou Metro Group Company, Ltd. (hereinafter referred to as the Metro Group), which has two branches under its jurisdiction, including the Zhengzhou Metro Group Company Ltd operation branch (hereinafter referred to as the Operation Branch), which is specifically responsible for the operation and management of the completed lines. The OCC was established under the operation branch. It was not only responsible for the scheduling of the metro lines, but also assumed the functions of an emergency command center. During the flood season, flood control teams should be present at metro stations.

According to the Regulations of Zhengzhou City Rail Transport, the safety responsibilities of the Metro Group include setting up a particular safety management agency; inspecting the lines under its jurisdiction and disposing of safety hazards in a timely manner; restraining behaviors that impede the safety of rail transit operations; reviewing engineering activities in the vicinity of rail transit lines to ensure that they do not affect safe operations; formulating comprehensive emergency response plans for emergencies and establishing emergency disposal mechanisms; executing the plans promptly when emergencies occur; and suspending rail transit operations when troubles seriously affect safe operations.

(2) System development

The China Railway Siyuan Survey and Design Group Company, Ltd. (hereinafter referred to as the chief design contractor) was the overall general contracting contractor for Metro Line 5. The Beijing Urban Construction Design and Development Group Company, Ltd. (hereinafter referred to as the parking lot design contractor) was the design contractor for the Wulongkou parking lot of Metro Line 5. The Power Construction Corporation of China (hereinafter referred to as the construction contractor) was the contractor for the construction of the Wulongkou parking lot fence. The Xinjiang Kunlun Engineering Consulting and Management Group Company, Ltd. (hereinafter referred to as the supervision contractor) was responsible for supervising the construction of the Wulongkou parking lot. The Zhengzhou City Engineering Quality Supervision Station (hereinafter referred to as the supervision unit) was responsible for executing the construction from the government side and issuing the opinion for project acceptance. The chief design contractor reviewed the engineering standards for the entire metro project; these standards included flood protection design standards. The parking lot design contractor undertook the design work for the Wulongkou parking lot on behalf of the general design contractor, under relevant legal requirements in China. 

The Construction Laws of the People’s Republic of China stipulate that the general design contractor can subcontract part of the project to a subcontracting design contractor that has the relevant qualifications, and the subcontracting design contractor is responsible to the general contractor; and if the project quality is not up to standard, or if there is a production accident, the general contractor is jointly and severally liable with the subcontracting design contractor. The supervision unit is a third party entrusted by the investor of the construction project to supervise the construction contractor. According to the Construction Laws of the People’s Republic of China, the supervision unit has the right to suspend construction upon finding that the construction quality is not up to standard. Under the guiding ideology of “strengthening local management,” local governments play an increasingly important role in actual emergency management. In response to natural disasters, such as heavy rainstorms, snowstorms, and typhoons, a professional natural disaster response and reduction committee will be established by the local government to provide proper guidance to the professional rescue forces.

#### 3.2.4. The Zhengzhou Government’s Emergency Control Structure

In the case of the fatal accident on Zhengzhou Metro Line 5, the Zhengzhou government’s emergency control structure contained the following important system components. 

(1) The city’s flood control headquarters, which was the most essential part of Zhengzhou government’s emergency control structure, as the command authority for flood control. (The command authority usually has an office in the Emergency Management Bureau. However, due to the highly specialized nature of flood control, the specific work of the office is often carried out by the water resources department.) 

(2) The Emergency Management Bureau, which was responsible for disaster management and reporting to the flood control command. 

(3) The Water Resources Bureau, which was responsible for reporting water warning information and preparing a flood and drought disaster defense plan. 

(4) The Urban Management Bureau, which organized relevant units to address urban flooding and issued urban flooding warning. 

(5) The Transportation Bureau, which supervised and guided the Metro Group’s safety inspection work and reported to the flood control office upon receiving information on metro danger. 

(6) The Urban and Rural Construction Bureau, which reviewed the design of flood prevention for various construction projects in the city.

(7) The Metro Group, which was in charge of the safe operation of the subway lines under its jurisdiction, for evacuating passengers in case of danger, and for reporting dangers to government departments.

(8) The Meteorological Bureau, which provided early warnings of rainstorms. 

**Figure 3:**
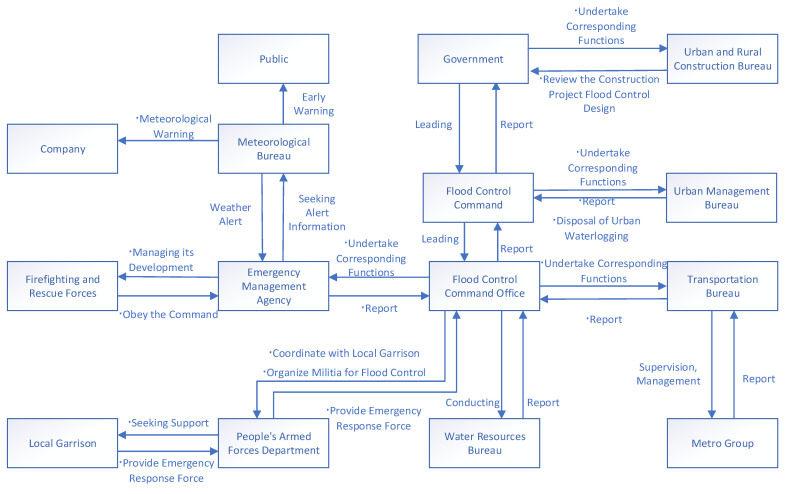
The Zhengzhou government’s emergency control structure.

The structure of public safety control during a heavy rainstorm in Zhengzhou is shown in Figure 3.

In 2018, following the reform of government agencies, many existing emergency response forces were integrated into the Emergency Management Bureau, which became the new emergency management agency. However, according to the Flood Control of the People’s Republic of China, the competent water administration departments of local governments at or above the county level are responsible for the organization, coordination, supervision, guidance, and other daily work of flood control in the administrative regions under the leadership of the governments at this level. Thus, in the emergency management of flood control in response to heavy rainfall, the water conservancy department is the central emergency department that is responsible for developing flood control planning, constructing flood control measures, and formulating flood control plans. The flood control command includes not only the heads of government departments, but also the heads of the local garrisons. The local garrisons carry out the orders of the command, together with the local fire rescue force. However, the highest commanders are the chief executives of the local governments.

#### 3.2.5. CAST Analysis for Zhengzhou Metro

Chinese metro trains are divided into A, B, and C types, according to the width of the trains. The A-type train is the most advanced type in China at present. It is used in Zhengzhou Metro Line 5. Theoretically, this type of train is much safer than the other types. However, in this accident, the A-type train lost power after water ingress, leaving passengers trapped on board. The official accident report did not conclude that there was a quality problem with the train. It concluded that the harshness of the environment at the time of the accident was beyond the designed capabilities of the train. In fact, it was not until several days after the accident that the water in the metro tunnel was completely emptied. On the one hand, the subway is a low-lying area under the ground, and water will naturally collect there. On the other hand, the subway can only rely on pumping machines to pump the water up to the ground. In Zhengzhou, there was a great deal of waterlogged water on the ground during the rainstorm, and the Zhengzhou metro was not equipped with strong pumping equipment, resulting in the slow removal of water in the metro with nowhere to drain. Eventually, a large amount of water caused the subway system to be paralyzed, and trapped passengers were waiting for emergency rescue in standing water. 

At 4:00 p.m. on 20 July 2021, the Metro Group did not start emergency response in a timely manner when water entered Metro Line 5 in many places. According to the emergency plan, there should have been a person in charge of the OCC to exercise emergency command. In fact, the relevant leaders of the Metro Group were not present in the OCC, resulting in a failure of the emergency command center functions of the OCC. The meteorological department had issued several red warnings for heavy rainstorm before the water filled the subway tunnels. However, the leadership of the Metro Group failed to conduct safety hazard checks on the subway lines, as required by the emergency plan. This indicated that the mental model of the leadership of the Metro Group was not sufficient to deal with heavy rainstorms and waterlogging. 

The mental model is a person’s persistent conceptual cognition of the world around them [65]. It showed that the safety education of the people in charge was inadequate and that the safety awareness of the people in charge was not sufficient for them to be aware of the huge challenges to the metro system that were brought about by the impending rainstorm. This observation leads to another question, i.e., who is responsible for ensuring that the people in charge are effectively educated with respect to safety? Apparently, there were no effective means to address the question within the Metro Group.

The “One Planning plus Three Systems” concept is one of the emergency management approaches that are implemented by the Chinese government. It requires that both government units and manufacturing enterprises establish reasonable emergency plans. Although the Metro Group had a corresponding emergency plan, it was not effectively implemented during the rainstorm. 

The implementation of an emergency plan relies on the effective functioning of the emergency response regulatory body. The emergency mechanism is usually understood to be a set of systems and methods that ensure the smooth and effective operation of an emergency response [66]. The emergency response mechanism of the Metro Group was not functioning well at the beginning of the accident, which led to a series of problems, such as a delayed emergency response and slowly reported disaster situations. Additional research is needed on building an effective emergency response mechanism.

The OCC dispatcher’s role was to direct the safe operation of the train. During the incident, the dispatcher ordered the train, which had already stopped at Beach Temple station, to continue its journey and called on the driver to reverse the train after the driver reported that the water was over the track surface and the train had stopped. This action caused the train to lose power at a lower point than the train’s original stopping position. Official incident reports attribute this result to bad decision-making by the dispatcher; however, this may well point to another problem: i.e., the lack of a proper monitoring system to provide dispatchers with adequate information, leaving them with only empirically determined solutions.

The Wulongkou parking lot is located on the north side of Wulongkou Road, covering an area of 13.9 hectares. It is an open-air subway train parking station that is connected to Metro Line 5 by a tunnel. According to the official accident investigation report that was released, it was from this tunnel that the flooded water gushed in. The collapse of the water retaining wall in the parking lot caused water to flood into the subway tunnel. The official accident report stated that the section of the water retaining wall was of substandard construction quality, that it was not constructed according to the drawings, and that part of the wall had almost no water-retaining function. 

**Figure 4:**
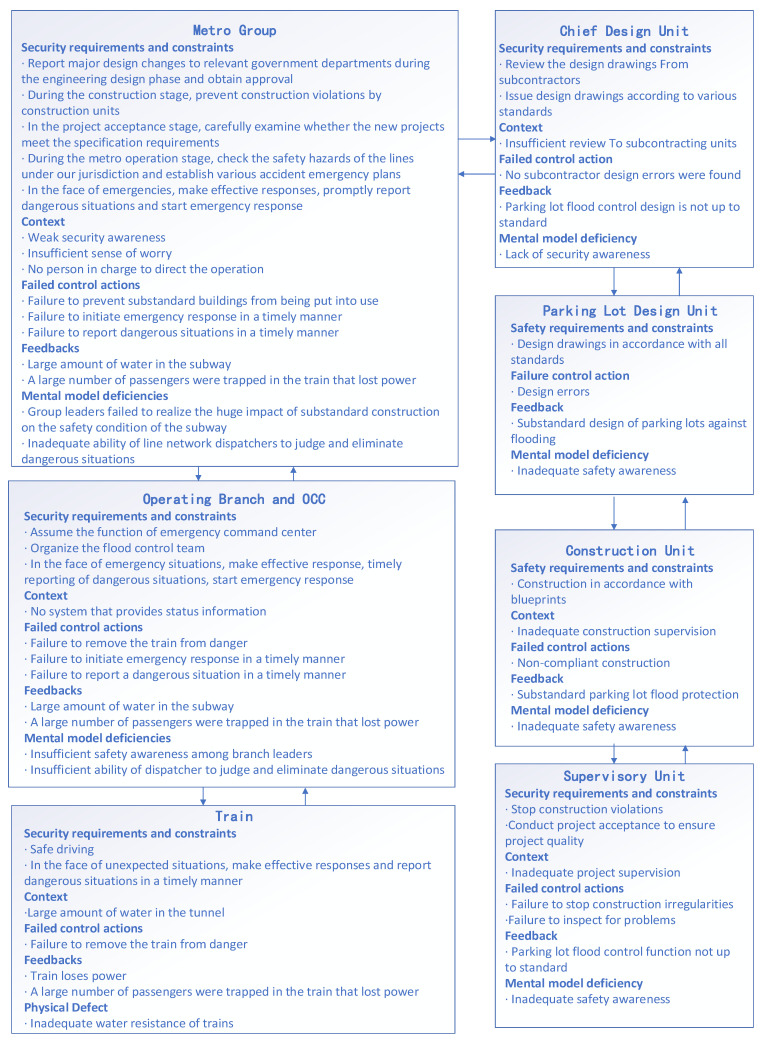
Zhengzhou Metro CAST Analysis.

After the investigation, it was found that the parking lot design contractor had changed the original design scheme during the engineering design stage; the construction contractor used the unexamined design drawings during the construction stage; the Metro Group agreed to the construction contractor’s illegal construction during the construction stage and failed to find that the water-retaining fence did not meet the specification requirements at the time of acceptance, a major hidden problem. This suggests that there was a lack of structure to control irregularities throughout the process from design to acceptance. In fact, the engineering supervision company should have acted as a control structure to eliminate violations, but for unknown reasons, the supervision company did not finish its job. This dereliction of duty led to safety hazards in the construction phase of Metro Line 5, which, combined with the special nature of the metro as a low-lying space, made emergency management much more difficult, due to substandard disaster prevention facilities The CAST analysis of Zhengzhou Metro’s emergency control structure is shown in Figure 4. The left side of Figure 4 is the system components when the metro is running, and the right side is the system components when the metro was being built.

#### 3.2.6. CAST Analysis of the Zhengzhou Government’s Emergency Control Structure

One of the characteristics of this heavy rainstorm disaster in Zhengzhou was the extremely strong short-term rainfall. The maximum daily precipitation on 20 July 2021, reached 624.1 mm, setting a new extreme record for rainfall observation in mainland China. Under the attack of the heavy rain, the urban drainage system of Zhengzhou was overwhelmed in a short time, and the metropolitan area was seriously flooded. The accident investigation report mentioned that Zhengzhou plans to build an urban drainage system in 2030 that can cope with 199 mm of precipitation in 24 h. This means that even if such a drainage system were created, it would not be able to cope with the extreme rainfall that occurred on 20 July 2021. The lack of a sufficient flooding capacity in Zhengzhou is a physical control deficiency. 

A major reason for this deficiency is that this extreme rainfall was much more severe than expected. Zhengzhou is in a northern region of China, with low precipitation. Based on experience, Zhengzhou does not need an overly strong drainage capacity. If someone had proposed an infrastructure system that could have met this flooding demand, it would have been rejected by city managers as wasteful. 

Another reason for the deficiency is that the urbanization process has been too fast. The built-up area of Zhengzhou city expanded from about 92 square kilometers in 1991 to about 1200 square kilometers in 2021, which was nearly 13 times the original size. A large amount of soil land that could absorb rainwater was covered with concrete building materials that lack water absorption ability, which reduced the drainage capacity of the city dramatically.

The Zhengzhou city government is the main body of this natural disaster emergency management. The components of the Zhengzhou government’s emergency control structure, except for the Metro Group, are all functional departments of the government, and these functional departments play their respective roles under the flood control command. The chief executive of the Zhengzhou government should be a controller, issuing orders in the emergency control structure. However, during this incident, there was no system component in the emergency control structure that could supervise the controller. This omission delayed the controller’s response to the disaster. The supervisor of the controller (the chief executive) had an important responsibility for improving the emergency control structure. The chief executive, as the top commander of the flood control command, should have been an active promoter of the emergency response. Nevertheless, after making a subjective judgment based on his personal experience that the storm would not be severe, he failed to implement the emergency deployment of the higher government and delayed the emergency response. These failures suggest that the mental model for the chief executive was deficient in responding to the disaster. 

The chief executive, as a government official, was deficient in disaster preparedness expertise. The chief executive had only assumed office shortly before the rainstorm, so he had limited familiarity with local conditions. This was another problem that was hidden by the flawed mental models. Although the chief executive responsibility system increases the smoothness of the emergency response mechanism, it places too much of the professional competence that is required in the hands of government officials who have limited professional competence. The chief executive system, which places government officials at the core of the emergency response mechanism, has become a double-edged sword in emergency management.

The Zhengzhou Emergency Management Bureau, the Zhengzhou Water Resources Bureau, and the Zhengzhou Urban Management Bureau, as essential components of the emergency management system, suffered from the same problem: i.e., insufficient vigilance against disasters. The Zhengzhou Emergency Management Bureau failed to implement the highest level of emergency response required by the emergency plan. After the meteorological department issued several red warnings, major dangers arose in the reservoirs outside the city, which seriously affected the control of the disaster situation. The responsibilities of the Zhengzhou Municipal Water Resources Bureau in the emergency management system included compiling the Zhengzhou Flood and Drought Disaster Prevention Plan, issuing early warning information about water dangers, and launching emergency responses to flood and drought disaster prevention. However, these three responsibilities were not effectively implemented. 

The Zhengzhou Flood and Drought Disaster Prevention Plan was not complete when the disaster occurred. There was a severe delay in the release of early warning information about water dangers, and in the initiation of an emergency response. As an important unit of urban flood control, the Zhengzhou city administration Bureau did not clearly assign the flood control responsibilities as set out in the Zhengzhou City Flood Control Emergency Plan; it did not issue early warning information to the public on the urban flood situation, nor did it report on urban flood control to higher authorities. 

The performances of these government departments can be summarized as a poorly functioning emergency response mechanism. The problem behind these performances was that the local government departments did not have sufficient experience in implementing an emergency response. Zhengzhou is in a northern region of China, with limited precipitation and an average annual rainfall of 640.8 mm, but 624.1 mm of rain fell on 20 July 2021. The last time a similar rainstorm disaster occurred in Henan Province was in 1975. Apparently, Zhengzhou’s emergency system did not have the appropriate disposal experience when the storm occurred. Although the Zhengzhou Urban and Rural Construction Bureau did not have important responsibilities in the emergency response process, its daily work had a huge impact on emergency management. Its failure to identify major problems (e.g., the actual location of the Wulongkou parking lot, which was lower than its present location, and the substandard construction of the water retaining fence) made it even more difficult for the metro system, which already had disadvantages in dealing with and withstanding the flooding. This failure highlighted the fact that the security control structure in Zhengzhou was focused on disaster prevention at the organizational management level and not sufficiently on the physical infrastructure level.

**Figure 5:**
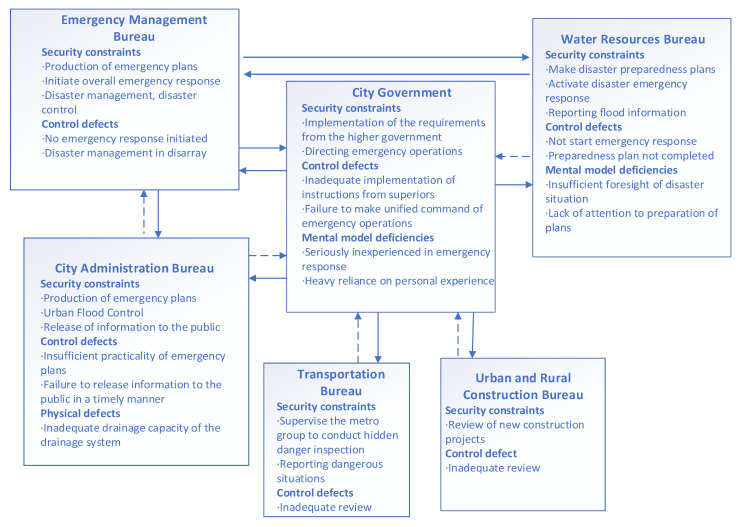
CAST analysis of the Zhengzhou government’s emergency control structure.

The CAST analysis of the Zhengzhou government’s emergency control structure is shown in Figure 5. The solid arrows represent effective information transmission and the dashed arrows represent invalid information transmission.

#### 3.2.7. Recommendations

The final step in a CAST analysis is to propose countermeasures based on the analysis results. Leveson noted that the purpose of accident analysis is not to determine who is responsible for an accident [61]. If an accident investigation is conducted solely to determine responsibility, the actual cause of the accident may be obscured by stakeholders. Because the responsibility for an accident is often attributed to an operator who directly caused the accident, further investigation into the cause of the accident may be prevented. For example, it may be difficult to determine out why the operator acted in a way that led to the accident. An accident investigation becomes meaningless if the actual cause of the accident is not identified, and if adjustments are not made to the safety control structure.

After our analysis of the heavy rainstorm disaster in Zhengzhou, Henan Province, the following recommendations are proposed.

(1) The role of expert committees in disaster response should be enhanced. The ability of emergency managers to understand information is extremely important in responding to disasters [67]. Apparently, professionals can more quickly identify the most valuable information and make adjustments based on this information when they are provided with sufficient amounts of disaster-related intelligence. In addition, some studies have indicated that in the process of responding to disasters, there is a lack of official supervision [68], and officials may initiate improper response measures that result in increased losses. After analyzing the accident process, we found that the chief executive, as an important controller in the emergency management system, had a great influence in determining the emergency action. However, the emergency management system lacked a component to correct the controller’s inappropriate decisions. Therefore, we recommend the establishment of an expert committee with a fixed number of members and providing such a committee with greater powers to make recommendations. When the meteorological department issues alerts to the government, it must also send the information to the expert committee. The expert committee should request that the local government expeditiously hold a disaster relief hearing, with the participation of the chief executive. If the chief executive of the local government does not pay attention to the impending disaster, the expert committee should have the power to report to the higher government. During the disaster response process, the expert committee should promptly put forward its suggestions.

(2) The relevant national standards should be updated. Due to the island effect of urban heat, atmospheric pollution, and other problems, heavy rainfall in urban areas is currently more frequent, and the amount of precipitation is increasing. Some Chinese scholars have pointed out that both extreme precipitation and precipitation days are increasing in China. The average increase in continuous extreme precipitation is 4.0 mm per decade [69], and the frequency and amounts of extreme precipitation in urban areas are on the rise [70]. It can be expected that extreme weather events, similar to the heavy rainstorm in Zhengzhou, will continue to occur in the future. The lack of sufficient flooding capacity in Zhengzhou is a microcosm of the existing national standards that are gradually becoming obsolete. The relevant standards need to be improved [71]. In addition, metro train-related standards need to be updated to avoid waterlogged tunnels that cause trains to lose power.

(3) Water storage facilities for subway tunnels should be constructed. In this incident, the only way to cope with the waterlogged Metro Line 5 after the waterlogged water broke through the retaining wall was to use sump pumps to pump the water to the surface. However, this method was clearly ineffective, as there was already a large amount of flood water on the ground. As a result, we propose building water storage facilities in the subway system, at a lower level than the subway tunnels, to accommodate the water that pours into the tunnels. As there is no place on the city streets to drain the water, a further suggestion is to build large urban underground drainage facilities and connect them to underground spaces, such as subway tunnels, to collect the water. These facilities can be based on the urban drainage systems that are already in place in developed countries, such Paris, London, and Tokyo. Practical considerations must be taken into account; for example, subsoil is an important factor affecting underground works [72]. In addition, care should be taken when constructing drainage facilities to keep them away from sandy layers, to avoid them being initially damaged during heavy rainfall. Such facilities can be integrated into sponge city construction, pursuant to a flood control policy that is strongly advocated by the Chinese central government [73]. The overall goal of this policy is to strengthen the construction of drainage systems in Chinese cities. It covers many elements, such as the green roof, runoff volume reduction systems, stormwater drainage systems, and outfall flood control systems.

(4) The supervision of lower-level governments by higher-level governments should be strengthened. Although the national and provincial systems played a role in both disaster warning and emergency support during this disaster emergency, the supervision work before the disaster was insufficient. The Zhengzhou municipal government did not initiate targeted deployment after the requirements were imposed at the higher level. To solve such problems, the lower levels of government should be ordered to come up with specific emergency response plans during work that is being supervised. The responsibility for emergency work should be assigned to individuals, so that the leaders of lower levels of government can feel pressure to act during and after the emergency.

(5) Emergency managers should be involved in reviews of construction projects. The levels of the disaster prevention capabilities of urban buildings have a great impact on emergency management actions. Excellent disaster prevention capabilities will effectively reduce emergency pressure. When disasters occur, managers of emergency operations can rely on these buildings in facilitating effective disaster response actions. In fact, buildings that do not meet flood control requirements have created a great problem for flood control. However, the city’s construction management has not addressed this problem. It is likely that reviews of building safety capacities are beyond their capabilities. In any case, inadequate building capabilities exacerbate the difficulties in the emergency management of disasters. Therefore, we propose that emergency management departments should join in the reviews, so that those responsible for disaster prevention have the authority to review building construction as part of urban disaster prevention. The unification of responsibilities for review and authorization can effectively enhance the various safety inspections of construction projects.

## 4. Discussion

Most of the current research on emergency management has focused on management structures [74], management methods [75], software systems [76], and conceptual theories [77]. There is less research on the role of disaster prevention facilities in the emergency management process. By analyzing the heavy rainstorm disaster in Zhengzhou, Henan Province, with the CAST method, we concluded that the construction of disaster prevention facilities can seriously impact the capability of the emergency management systems. It is difficult to compensate for deficiencies in disaster prevention facilities by pure management methods. In the fatal accident of Zhengzhou’s Metro Line 5, if there had been no quality problem in the retaining wall of the parking lot; if the subway tunnel had had good drainage facilities; if the train had not lost power after entering the water; and if the dispatcher had had equipment that could have helped in judging the situation, then the management problems (such as the lag in emergency response and the absence of command of the emergency work) may have been remedied. 

Even if there were no management problem, when faced with the situation which flooded water poured into the tunnel in large quantities, all the emergency command center could do was to organize a rescue, rather than disposing of the water to reduce its impact on train operation. As we have seen, the construction of disaster prevention facilities for emergency systems is an important factor in improving emergency management capabilities. The lack of disaster prevention facilities is essentially a problem that has resulted from rapid urban development. 

The existing disaster prevention facilities were not sufficient to deal with the disaster threats facing the huge city, and new disaster prevention facilities are still under construction. It is impossible to build new disaster prevention facilities because of the existing buildings in the old city. Since 2013, China has proposed building sponge cities to address this issue [78]. However, that proposal involves urban planning, and the impact of urbanization on emergency management requires further research.

The CAST analysis method was originally designed to analyze socio-technical systems. However, in this paper, it has provided excellent results in analyzing purely social systems at the government level. Based on the official accident report and relevant information obtained in our investigations, we analyzed accidents and made recommendations that are not found in official accident reports. Although the CAST method explores the causes of an accident from the perspective of system science, it does not attribute the cause of an accident to a specific operator. However, in analyzing social systems, human error cannot be hidden. 

We believe people (controllers and other individuals) should be considered as components in the control loop of the system, according to their roles in the system. In searching for the systemic causes of the failure by the human component, researchers can uncover problems with the component itself. This paper proved that the CAST method can further extend the scope of research to purely social systems, such as various management systems, if humans are considered as system components. If we consider people as system components and analyze their internal problems, only mental models are studied. In future research, more intrinsic reasons for the failure of human components can be identified to expand on the causes of accidents and to enhance the analysis perspectives of the CAST method. Another purpose of Leveson’s CAST method is to use official accident reports to find causes of accidents that are overlooked by official investigators. However, in practice we have found that it is not enough to analyze accident reports. More details of an accident need to be investigated by the researchers themselves. Only by mastering these details can researchers discover hidden causes of an accident. This conclusion has been pointed out by other scholars [63]. Different researchers may obtain different information through their investigations and reach different conclusions about the causes of accidents. However, they can also be misled by certain information and draw wrong conclusions. Therefore, a possible next step in the development of CAST methods is to provide a process to help researchers in identifying whether information they obtain is valid.

A shortcoming of this study is that the responsibilities of some departments in the Zhengzhou government’s emergency control structure cannot be clearly understood from the available information. Most of the current governmental emergency plans are non-public, which led us to use accident investigation reports (rather than specific emergency plans) as important materials for this study. The central departments involved in emergency work can identify their responsibilities from the government’s official website. However, the roles of some auxiliary departments can only be known from an accident investigation report. Therefore, the details of the Zhengzhou government’s emergency control structure, which were obtained in this study, may be further reviewed.

## 5. Conclusions

By analyzing an accident that followed a typical natural disaster, this study proved that the CAST method is effective and reliable in analyzing a social system such as an emergency management system. However, some conceptual innovation is required when analyzing social systems. We used the CAST method to study the management of the Zhengzhou Henan heavy rainstorm disaster and put forward some suggestions. However, this heavy rainstorm event in Zhengzhou required further in-depth analysis. By summarizing the lessons of the accident, we provided further perspectives for future emergency management research. This article is a preliminary discussion in that regard.

Although many scholars have made various suggestions for improving China’s emergency management system, the government must verify their effectiveness through evaluation and practical applications. Fortunately, over the past few decades, the Chinese government has focused on optimizing its emergency management methods. Government agencies can use the CAST method to summarize the problems exposed during post-incident exercises, which will help them in identifying future improvements that are required.

Official investigations of accidents focus on determining who was responsible and imposing penalties on those who were responsible. However, we recommend the use of the CAST method in the investigation process, as it can help in understanding the accident mechanisms and contribute to the prevention of similar accidents.

## Figures and Tables

**Table 1:** The timeline of the events in the Metro Line 5 fatal incident.

Approximate Time	Event
15 July 2021	The Henan Provincial Meteorological Bureau issued a warning to the Henan provincial party committee and the provincial government.
16 July 2021	The Henan provincial party committee and thepProvincial government initiated flood control deployment.
18 July 2021	The working group of the State Council guided flood control work.
20 July 2021	The meteorological department issued a second red alert for heavy rainfall; the municipal government and the other levels of government began to check the flood control work.
20 July 2021 16:00	Metro Line 5 leaked in many places.
20 July 2021 17:00	A large amount of waterlogged water poured into the metro tunnel.
20 July 2021 17:35	The train stopped temporarily at the Beach Temple station.
20 July 2021 17:46	The train continued to move.
Unknown time point	The train lost power.
20 July 2021 18:05	Attendants tried to evacuate passengers.
20 July 2021 18:40	Evacuation was aborted, and the passengers returned to the train; hundreds of passengers were trapped.

**Table 2:** The security constraints of various levels.

Level	Security Constraints
Operational level	The drivers drive trains according to regulations and report any dangerous situations to the OCC on time. The OCC issues orders to drivers and serves as the metro emergency command center.
Company level	The company complies with relevant laws and regulations. The company formulates appropriate emergency plans and ensures that employees are familiar with the contents of the plans. The company conductd safety inspections of the subway lines. The company ensures the safe evacuation of train passengers. The company promptly reports any dangerous situation to the government transportation department.
Government level	The local government formulates various emergency plans. The local government issues early warnings to the public. The local government announces the interruption of daily production and life. The local government directs professional emergency forces. The local government supervises various security efforts within its jurisdiction.

## Data Availability

The source of climate data in this article may be found at http://www.cma.gov.cn/2011xwzx/2011xqxxw/2011xqxyw/202204/t20220408_4743526.html (accessed on 18 May 2022). The full text of the official accident investigation report may be found at https://www.mem.gov.cn/gk/sgcc/tbzdsgdcbg/202201/P020220121639049697767.pdf (accessed on 3 March 2022).
